# Neonatal Venous Thromboembolism

**DOI:** 10.3389/fped.2017.00136

**Published:** 2017-06-06

**Authors:** Kristina M. Haley

**Affiliations:** ^1^Pediatric Hematology/Oncology, Oregon Health & Science University, Portland, OR, United States

**Keywords:** developmental hemostasis, neonatal thrombosis, thrombophilia, neonatal venous thromboembolism, renal vein thrombosis

## Abstract

Neonates are the pediatric population at highest risk for development of venous thromboembolism (VTE), and the incidence of VTE in the neonatal population is increasing. This is especially true in the critically ill population. Several large studies indicate that the incidence of neonatal VTE is up almost threefold in the last two decades. Central lines, fluid fluctuations, sepsis, liver dysfunction, and inflammation contribute to the risk profile for VTE development in ill neonates. In addition, the neonatal hemostatic system is different from that of older children and adults. Platelet function, pro- and anticoagulant proteins concentrations, and fibrinolytic pathway protein concentrations are developmentally regulated and generate a hemostatic homeostasis that is unique to the neonatal time period. The clinical picture of a critically ill neonate combined with the physiologically distinct neonatal hemostatic system easily fulfills the criteria for Virchow’s triad with venous stasis, hypercoagulability, and endothelial injury and puts the neonatal patient at risk for VTE development. The presentation of a VTE in a neonate is similar to that of older children or adults and is dependent upon location of the VTE. Ultrasound is the most common diagnostic tool employed in identifying neonatal VTE, but relatively small vessels of the neonate as well as frequent low pulse pressure can make ultrasound less reliable. The diagnosis of a thrombophilic disorder in the neonatal population is unlikely to change management or outcome, and the role of thrombophilia testing in this population requires further study. Treatment of neonatal VTE is aimed at reducing VTE-associated morbidity and mortality. Recommendations for treating, though, cannot be extrapolated from guidelines for older children or adults. Neonates are at risk for bleeding complications, particularly younger neonates with more fragile intracranial vessels. Developmental alterations in the coagulation proteins as well as unique pharmacokinetics must also be taken into consideration when recommending VTE treatment. In this review, epidemiology of neonatal VTE, pathophysiology of neonatal VTE with particular attention to the developmental hemostatic system, diagnostic evaluations of neonatal VTE, and treatment guidelines for neonatal VTE will be reviewed.

## Introduction

Venous thromboembolism (VTE) is increasingly recognized and diagnosed in the neonatal population. The neonatal patient is at risk for VTE due to a combination of factors that can easily tip the hemostatic balance toward thrombosis such as invasive medical procedures, systemic inflammation, central venous catheters (CVCs), fluid fluctuations, and infection. In addition, the developing hemostatic system may be more vulnerable to disruptions and may more easily shift toward thrombosis. Improved attention to and recognition of VTE symptoms is also resulting in an increased incidence of neonatal VTE. Data are still limited with regard to optimal diagnosis and management of VTE as well as prevention of VTE in the neonatal population.

## Epidemiology

Venous thromboembolism is a relatively rare disease in the pediatric age group. However, VTE is increasingly considered and diagnosed in pediatric patients. Canadian registry data from the early 1990s indicated a VTE incidence of 5.3 per 10,000 hospital admissions and an overall incidence of 0.07 per 10,000 children (1). Subsequent studies have highlighted the increasing incidence in the last decade, with as much as a 70% increase of VTE incidence from 2001 to 2007 (2). In a retrospective cohort study of the Pediatric Health Information System (PHIS) database, the incidence of VTE increased from 34 per 10,000 hospital admissions in 2001 to 58 per 10,000 hospital admissions in 2007. For PHIS database, patients less than 28 days old, the VTE incidence was approximately 75 per 10,000 hospital admissions (2). The distribution of VTE across the pediatric age group has been reported to be bimodal, with one spike in incidence in the neonatal period and a second spike in the adolescent period (1, 2). However, some studies have suggested that this bimodal distribution disappears when the data are standardized for a number of discharges or a number of admissions, and instead show an overall increased incidence of VTE with age (3, 4). Regardless, the incidence of VTE in the neonatal population remains high and is increasing (2, 5) and warrants continued investigation with regard to diagnosis, prevention, and management.

## Developmental Hemostasis

The neonatal hemostatic system is both quantitatively and qualitatively distinct from that of an older child or adult. The term “developmental hemostasis” has been applied to the period of time when the neonatal hemostatic system exists in an evolving balance of pro- and anticoagulant factors (6). Neonatal levels of factor VII, fibrinogen, and alpha-1 antitrypsin are similar to older children and adults, but all other pro- and anticoagulant proteins are deficient in neonates on day of life one and reach adult values by around 6 months of age (6). Neonatal levels of the anticoagulants, ATIII, protein C, and proteins S are similar to the levels of individuals with heterozygous deficiencies (6, 7). Differences in the hemostatic system are magnified in premature infants; yet, hemostatic system maturation is accelerated as compared to term infants (8). Despite the decreased pro-coagulant levels and the decreased anticoagulant levels, the healthy newborn is believed to exist in a hemostatic balance, neither prone to hemorrhage nor thrombosis (7). However, the balance is delicate and can be easily tipped in either direction (7).

## Risk Factors

### Acquired Risk Factors

The neonatal hemostatic system can be tipped toward thrombosis by a variety of acquired risk factors, and greater than 95% of neonatal VTE is associated with at least one clinical risk factor (5, 9) (Table 1). The most common clinical risk factor is the presence of a CVC (5, 9–13). A prospective study of neonates who underwent CVC placement indicated a 13% incidence of CVC-associated VTE (14), while another evaluating only umbilical catheter revealed a higher incidence of 22% (15). A recent systematic review of the literature noted that risk factors for CVC thrombosis development are not reliably documented across studies, however; birth weight, gestational age, prolonged catheter duration (>6 days), umbilical venous catheter (UVC) mal-placement, and the addition of blood products to UVC infusions were all highlighted as risk factors for CVC-related thromboses (5, 16).

**Table 1:** Risk factors for venous thromboembolism (VTE) development in neonates.

Maternal risk factors	Neonatal risk factors	Risk factors specific to central venous catheter (CVC)-related VTE
Infection	CVC	Low birth weight
Placental disease	Sepsis	Prematurity
Diabetes mellitus	Congenital heart disease	Prolonged catheter duration (>6 days)
Hypertension	Perinatal asphyxia	Umbilical venous catheter (UVC) mal-placement
Pre-eclampsia	Dehydration	Addition of blood products to UVC
Dyslipidemia		Mechanical ventilation
Metabolic syndrome		Surgery
Antiphospholipid antibody syndrome		
Emergent C-section		
Premature rupture of membranes		
Inherited thrombophilia		

In addition to CVCs, sepsis, mechanical ventilation, perinatal asphyxia, congenital heart disease, and dehydration are recognized risk factors for neonatal VTE (11, 13, 17). The contribution of maternal risk factors to neonatal VTE development is not as well studied, however; infection, placental disease, diabetes mellitus, hypertension, pre-eclampsia, dyslipidemia, metabolic syndrome, antiphospholipid antibody syndrome, inherited thrombophilia, emergent Cesarean section, and premature rupture of membranes have been associated with increased risk of neonatal VTE (18). At least one or more maternal risk factor for neonatal VTE has been found in up to 56% of neonates with venous thrombosis (18). Risk factors for renal vein thrombosis (RVT) are similar to other neonatal VTE risk factors and include prematurity, maternal diabetes, dehydration, infection, perinatal asphyxia, and umbilical vein catheter (13, 19). The pathophysiology is suggested to result from impaired renal perfusion under the listed clinical circumstances, which leads to vasoconstriction and subsequent impaired venous blood flow which puts the postglomerular circulation at increased risk for thrombosis (20).

### Inherited Thrombophilia

The role of inherited thrombophilic risk factors in neonatal VTE development is poorly defined (21). A recent systematic review analyzed 13 publications from 2008 to 2014 evaluating the role of inherited thrombophilia in neonatal VTE development and treatment. The authors concluded that neonatal VTE is multifactorial and clinical risk factors weigh more heavily on the prothrombotic scale than inherited thrombophilia, particularly in CVC-associated VTE (9). In an earlier study, the overall prevalence of inherited thrombophilia in neonates with VTE was not different than that of the healthy population, concluding that screening neonates with VTE for inherited thrombophilia was not necessary (12). In contrast, in another study of catheter-related VTE, 15 of 18 infants with VTE had at least one inherited thrombophilia (10). Further, in an Italian registry of neonatal VTE, an inherited thrombophilia was found in 33% of infants with an “early-onset” VTE (VTE in the first day of life) (18). While inherited thrombophilia appears to be present in some neonates with VTE, both CVC related and not, the role of inherited thrombophilia testing in neonates with VTE remains up for debate as it does not appear, at this time, to influence type or duration of treatment (9).

## Clinical Presentation

Neonatal VTE most commonly occurs in the hospital, in the neonatal intensive care unit, as a reflection or effect of more significant illness. However, neonatal VTE can be the admitting diagnosis for neonates, as in RVT or cerebral sinus venous thrombosis (CSVT). Signs and symptoms of VTE in neonates are dependent upon the VTE location. A recent publication detailing data from a multicenter network of Italian investigators noted that of the 75 neonatal thromboses, 57 (76%) were associated with symptoms at diagnosis. For the VTE cases, 31/41 thromboses were associated with symptoms such as edema (50%), limb discoloration (34%), abdominal mass (10%), and central venous line dysfunction (7%) (18). The remaining thromboses were found incidentally on imaging obtained for other reasons (18). Symptoms of RVT include hematuria, abdominal mass, and/or thrombocytopenia. CSVT symptoms include seizures, apnea, agitation, decreased alertness, and symptoms of infection (22). The non-specific symptoms of respiratory failure, apnea and bradycardia, thrombocytopenia, and persistent bacteremia have also all been reported as symptoms of VTE (11).

Premature infants are more likely to be diagnosed with a VTE than term infants (21). In the above mentioned Italian registry study, preterm neonates accounted for 71% of thromboses (18). Timing of diagnosis is related both to gestational age and to site of thrombosis. In a registry of pediatric hospitals in Germany, venous thrombosis diagnosis at day of life 11 or 12 was more common than diagnosis at birth (17). RVT was more commonly diagnosed soon after birth in term infants but later (day 8 of life) in premature infants (17). In the Canadian registry, spontaneous RVT was more common in term infants while all other thromboses were found across gestational ages (11). Differences in timing of VTE presentation between premature and term neonates are likely related to acquired VTE risk factors related to underlying illness and intensive care.

### Diagnostic Imaging

Imaging modalities employed to diagnose VTE in neonates include ultrasound, venography, computed tomography (CT), and magnetic resonance imaging (MRI). Ultrasound is the most common imaging modality employed to diagnose neonatal VTE, although venography is considered the reference standard for diagnosis of VTE (23). In the Canadian VTE registry study, Doppler ultrasound was used to diagnose 67% of the venous thromboses (11). Similarly, Doppler ultrasound was the most common imaging modality in the German VTE registry study (17). The availability of Doppler ultrasound as well as its non-invasive nature makes it an attractive diagnostic modality. However, it is operator dependent and its sensitivity and specificity decline when evaluating intrathoracic vessels as well as the iliac vessels or if the patient is edematous or has interfering skin abnormalities (23). A prospective study aimed at determining the incidence of asymptomatic venous thromboses associated with UVCs found that Doppler echocardiography was less sensitive than contrast venography (24). Further, the Prophylactic Antithrombin Replacement in Kids with ALL treated with Asparaginase study recommended a combination of ultrasound and venography to investigate upper extremity, line associated VTE (25). Venography is not often employed, though, given its invasive nature, technical demands, and radiation exposure (23). CT or MRI can be employed to evaluate the intrathoracic venous system for thromboses (26). A reasonable approach may be to start with ultrasound, and if negative and clinical suspicion remains high, then pursue evaluation with MRI or CT depending on diagnostic modality availability and contributing clinical factors.

## Treatment

Treatment of neonatal VTE cannot simply be extrapolated from recommendations for adult VTE as the neonatal hemostatic system, the neonatal vascular system, and neonatal co-morbidities create a delicate balance of hemorrhage and thrombosis. The severity of the thrombosis, the possibility of organ or limb impairment, the presence of comorbidities such as congenital heart disease, and the bleeding risk all influence the decision to treat or to observe (27). Randomized trials evaluating type and duration of treatment are lacking in the neonatal population, and treatment decisions are largely based on consensus evidence-based guidelines (28). The CHEST guidelines provide recommendations for a variety of thrombotic complications in the neonatal population, including RVT and CVC-associated thromboses (28). The CHEST guidelines will not be extensively reviewed here as they are accessible through online journals.

Antithrombotic therapy is aimed at reducing the risk of extension or embolization, to reduce the risk of recurrence, and to reduce the risk of postthrombotic syndrome (PTS) (29). Duration of anticoagulation is generally 3 months for provoked thromboses and may be up to 6 months for idiopathic thromboses (26). The anticoagulants most commonly used in treatment of neonatal VTE include unfractionated heparin (UFH) and low molecular weight heparin (LMWH). Developmental hemostasis, differences in drug metabolism, and unique comorbidities must weigh into the choice of anticoagulant. Both UFH and LMWH require higher doses in neonates than older children and adults to achieve therapeutic levels. A prospective study, which included full term and premature neonates, found LMWH to be a safe and effective form of anticoagulation (30). The advantages of LMWH include subcutaneous administration, more predictable pharmacokinetic profile, minimal monitoring requirements, and less bleeding risks (27). The advantages of UFH include potentially easier reversibility with protamine. A recent Cochrane review of heparin (both UFH and LMWH) for the treatment of thrombosis in neonates found no eligible publications for inclusion in their review and concluded that there are no trials to recommend or refute the use of heparin for treatment of neonates with thrombosis (31).

Vitamin K antagonists (VKAs) are difficult to use in the neonatal period owing to a variety of factors such as frequent monitoring, lack of liquid formulation, and naturally low levels of vitamin K dependent factors (27). In addition, formula is supplemented with vitamin K, perhaps negating some of the effects of VKA and breast milk is deficient in vitamin K, perhaps putting the breastfed infant at greater bleeding risk (27). A single randomized control trial comparing LMWH and UFH followed by VKA has been conducted and demonstrated that LMWH was effective for treatment of VTE and was not inferior to UFH/VKA in pediatric patients. Neonates were not included in the study (32). VKA treatment is generally not favored in the neonatal population, and either UFH or LMWH are more commonly employed. None of the direct oral anticoagulants has been approved in pediatric populations, however; studies are ongoing in the pediatric population for these new anticoagulants (33).

### Drug Dosing and Monitoring

Therapeutic monitoring of UFH is recommended with a goal anti-Xa level of 0.35–0.7 units/mL (28). It is suggested that UFH boluses should not be greater than 75–100 units/kg and should be avoided in those children where a significant bleeding risk exists (28). CHEST guidelines recommend starting the initial infusion at 28 units/kg per hour for infants but individual risk factors should be considered when choosing initial dosing (28). Neonatal dosing of LMWH is higher than older children and adults, and frequently doses of 1.5–2 mg/kg twice daily are needed to get into the therapeutic anti-Xa range of 0.5–1 units/mL (28, 34–36).

### Thrombolysis

Thrombolytic therapy employs different agents [tissue plasminogen activator (tPA), streptokinase, urokinase] to convert plasminogen to plasmin, which ultimately cleaves fibrinogen and fibrin to fibrinogen/fibrin-degradation products (27). The decreased plasminogen concentration in neonates may decrease the efficacy of these agents, though (27). On the other side, the delicate neonatal cerebral vasculature and immaturity of the hemostatic system may predispose the infant to bleeding complications. A retrospective review of thrombolysis from 1964 to 1995 identified that intracranial hemorrhage occurred in 1/83 term infants and 11/86 preterm infants receiving tPA (37). In another review of 16 neonates treated with tPA, 7 had complete resolution of their thrombosis while 7 had partial resolution. One neonate died while receiving tPA from massive intracranial hemorrhage, but tPA was given despite severe thrombocytopenia in this patient, which was thought to be a confounding factor in the patient’s bleeding (38). Current consensus guidelines recommend against thrombolysis therapy unless the thrombosis is life-, limb-, or organ-threatening (28). If thrombolysis is used, tPA is the recommended agent, and plasminogen administration (through transfusion of fresh frozen plasma) is recommend prior to beginning thrombolysis (28).

## Morbidity/Mortality

### Mortality

Data regarding morbidity and mortality of VTE in neonates are lacking as follow-up in the majority of registry studies is short. In the Canadian registry, mortality in neonates with RVT was 5%, with other venous thrombosis was 18%, and with arterial thrombosis was 21%. However, all deaths were not directly attributable to the thromboses (11). The mortality rates for both aortic and right atrial/superior vena cava thromboses were 33% (11). In further analysis of the outcomes in the Canadian registry, for children 1 month to 18 years, the all-cause mortality was 16% with a thrombosis-related mortality of 2.2% (39). In the German registry study, 9% (7/79) of neonates with thromboses died, with three of the deaths related to thrombosis (17).

### Recurrence

Recurrence rates are also difficult to determine accurately due to short follow-up times in most studies. The recurrence rate in the Canadian registry for children >1 month was 8.1% (39). The role of inherited thrombophilia on recurrent VTE is variable. Some studies suggest that the most important risk factor of recurrent thromboses is the presence of a clinical risk factor or the recurrence of the original clinical risk factor (12). However, other authors suggest that the presence of inherited thrombophilias, especially in combination, are risk factors for recurrent thrombosis and thus advocate their screening (40–42). Guidelines regarding the implementation of pharmacologic prophylaxis are lacking, and thus as no treatment change is currently recommended due to the presence of an inherited thrombophilia, testing for an inherited thrombophilia should be performed on an individual basis or, ideally, in the context of a clinical study.

### Postthrombotic Syndrome

Postthrombotic syndrome is a chronic complication of deep vein thrombosis and is characterized by chronic venous insufficiency. PTS results from a combination of residual thrombus causing obstruction and secondary valvular reflux (43). The data on neonates are lacking owing to variability in duration of follow-up in most of the studies and registries. In a retrospective study of children with upper extremity VTE, 16% of neonates developed mild PTS with collateral vein formation and increased extremity circumference. Lack of clot resolution and extension of the clot were identified as risk factors for PTS development (44). In the Canadian registry, PTS was diagnosed in 12.4% of the children with VTE (39). Data support the use of thrombolytic regimens in treatment of acute lower extremity DVT to reduce the risk of PTS in older children, but data are lacking in neonates (45).

### Heparin-Induced Thrombocytopenia (HIT)

Heparin-induced thrombocytopenia is a drug-induced, immune-mediated thrombocytopenia that is associated with the potential for serious thrombotic complications (46). The thrombocytopenia is typically moderate and the bleeding risk is low, however; the thrombosis risk is high (47). There is little literature on the development of HIT in the pediatric population, but the incidence is likely lower in children than in adults (46). The lower incidence is thought to be secondary to age-dependent differences in both the coagulation and immune systems (46). A recent review of the pediatric HIT literature reported an incidence between 0 and 1.7% in the neonatal subpopulation of anti-PF4/heparin antibodies but no cases of neonatal HIT (47). Further studies are needed to better understand HIT in the neonatal population. Given its significant deleterious outcomes, if suspected, all heparin should be stopped and a non-heparin alternative, such as a direct thrombin inhibitor (DTI), should be started until HIT is ruled out (48). Argatroban is the only DTI that has been prospectively studied in pediatric patients with HIT, and dosing guidelines are now included in the prescribing information (49, 50).

## Conclusion

Venous thromboembolism in the neonatal population is a distinct pathophysiological entity, requiring age-specific therapy and careful follow-up. Long-term follow-up studies are needed to more fully understand the impact of neonatal VTE diagnosis on patients as they age. A few special circumstances may require heightened attention such as patients who need long-term or repeated central venous access, the risk of PTS in the growing neonate, and the risk of recurrence for females who someday require estrogen therapy. There are many unknowns in the realm of neonatal VTE, which creates an excellent opportunity for research and investigation to improve our understanding of risks, treatments, and long-term management. Long-term registry data are needed in order to follow neonates who develop VTE more closely to obtain better information on morbidity and mortality acutely and chronically.

## Author Contributions

KH performed the literature review and wrote the manuscript.

## Conflict of Interest Statement

The author declares that the research was conducted in the absence of any commercial or financial relationships that could be construed as a potential conflict of interest.
